# Efficacy and Safety of Resveratrol in Type 1 Diabetes Patients: A Two-Month Preliminary Exploratory Trial

**DOI:** 10.3390/nu12010161

**Published:** 2020-01-06

**Authors:** Ali Movahed, Pema Raj, Iraj Nabipour, Marzieh Mahmoodi, Afshin Ostovar, Mohammadreza Kalantarhormozi, Thomas Netticadan

**Affiliations:** 1 Department of Endocrine and Metabolic Diseases, The Persian Gulf Tropical Medicine Research Center, Bushehr University of Medical Sciences, Bushehr 987514763448, Iran; inabipour@gmail.com (I.N.); mahmoodi.marzieh@gmail.com (M.M.); afshin.ostovar@gmail.com (A.O.); m.kalantarhormozi111@yahoo.com (M.K.); 2 Canadian Centre for Agri-Food Research in Health and Medicine, Winnipeg, MB R2H 2A6, Canada; praj@sbrc.ca; 3 Department of Physiology and Pathophysiology, University of Manitoba, Winnipeg, MB R3E 0J9, Canada; 4 Agriculture and Agri-Food Canada, Winnipeg, MB R3T 2M9, Canada

**Keywords:** resveratrol, type 1 diabetes, hyperglycemia, hemoglobin A1c, oxidative stress

## Abstract

Resveratrol has been reported to be beneficial against diabetes complications. The objective of this study was to evaluate the efficacy of resveratrol in decreasing hyperglycemia in patients with type 1 diabetes (T1D) by a preliminary investigation designed as an exploratory clinical trial. Thirteen patients with T1D from both the sexes participated in this trial. All patients received resveratrol in 500 mg capsules, twice daily for 60 days. Bodyweight, fasting blood sugar (FBS), hemoglobin A1c (HbA1c), insulin, homeostasis model of assessment for insulin resistance (HOMA-IR), homeostasis model of assessment for β-cell function (HOMA-β), and markers of liver and kidney damage, inflammation, and oxidative stress were measured before the intervention, at 30 days and at 60 days. Resveratrol supplementation for 60 days significantly decreased FBS and HbA1c in comparison with the baseline values. Resveratrol treatment also resulted in a decrease in the level of a marker for oxidative stress, malondialdehyde, and an increase in total antioxidant capacity in T1D patients. Insulin, HOMA-IR, HOMA-β, and markers of liver and kidney function and inflammation were not significantly affected by resveratrol treatment. Overall, the results showed that 60 days of resveratrol supplementation exerted strong antidiabetic and antioxidant effects in patients with T1D.

## 1. Introduction

Type I Diabetes (T1D) is an autoimmune disease-derived disorder wherein there is a lack of a sufficient level of insulin to regulate blood glucose, which leads to hyperglycemia and subsequent organ damage [1]. In T1D, dysfunctional pancreatic β-cells contribute to an absolute deficiency of insulin and debilitating heterogeneous diabetic complications. The incidence of T1D is increasing at an alarmingly high rate around the globe. It has been estimated that 387 million people are living with diabetes mellitus worldwide. T1D is found in approximately 5% to 10% of total diabetes mellitus patients [2]. Diabetes-induced abnormalities are the main cause of death in those who are diagnosed with T1D [1]. T1D has also been recognized to occur in children and adolescents more frequently. The recommended mainstay therapy for T1D involving intensive or conventional insulin treatment coupled with continuous blood glucose monitoring is still plagued by unfavorable side effects [3]. The effectiveness of insulin therapy may often prove counterproductive as it can also lead to hypoglycemic episodes [3]. Treatment-induced hypoglycemia is known to negatively impact the patient’s adherence to insulin treatment [4,5]. On the other hand, it is also recognized that T1D patients may not always achieve targeted glycemic levels, even with intensive insulin treatment [4]. Abnormal weight gain and higher cardiovascular risk are some of the complications associated with recommended insulin treatment. This subsequently affects the benefits of glycemic regulation in T1D patients [5,6,7].

There is an unmet need to effectively manage hyperglycemia in conjunction with the use of insulin in T1D patients [3,8]. Insulin treatment alone may not be sufficient to avoid all diabetes-related complications [3,8]. In this regard, new adjunctive therapies may prove beneficial for T1D patients in terms of achieving normal glycemic levels and preventing macro- and microvascular disease [3]. Better glycemic status and prevention of weight gain and hypoglycemia with reduced insulin dosage are the desirable outcomes expected from a new add-on therapy for T1D [9,10]. In view of the drawbacks of current therapy, potential add-on therapies have been explored for managing T1D. Incidentally, glucagon-like peptide 1 analogs/receptor agonist and sodium–glucose cotransporter 2 inhibitors are currently being investigated extensively as they are useful in controlling hyperglycemia in Type 2 Diabetes (T2D) patients. There have been some promising initial results in T1D patients with these drugs as well, suggesting the potential for the addition of new therapies to the current insulin-based therapy in T1D patients. However, it should be noted that these agents have not been approved for clinical use yet in T1D patients. In this context, food-derived compounds have also been shown to have beneficial effects in T1D. One such compound is resveratrol, which is found predominantly in grapes, peanuts, and berries. Preclinical studies have shown strong antihyperglycemic effects in different animal models of T1D. However, there is no evidence yet about the efficacy of resveratrol in providing benefits to patients with T1D.

Accordingly, we sought to examine this possibility by conducting an exploratory trial investigating the safety and efficacy of resveratrol in T1D patients.

## 2. Materials and Methods

### 2.1. Study Design and Subjects

**Figure 1:**
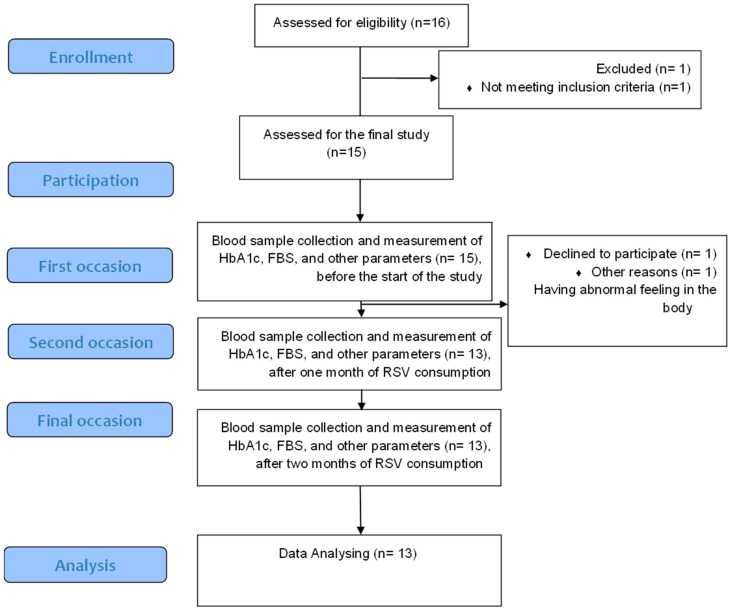
Consort flow diagram of the study.

This preliminary study was designed as an exploratory, single-center, two-month investigation to evaluate the efficacy and safety of resveratrol in T1D patients. The study was approved by the Medical Ethics Committee (IR.BPUMS.REC.1396.3.) of Bushehr University of Medical Sciences, Iran. The study also complied with the Declaration of Helsinki and the International Conference on Harmonization/Good Clinical Practice Guidelines. The project is registered with the Clinical Trial Registry of Iran (registration no: IRCT201710108129N11). Informed consent was obtained from each patient at the time of enrolment and continued as a process throughout the investigation. In addition, the patients had free medical care and consultation during the study period, especially in the case of any adverse reaction or complications. Thirteen patients with T1D, aged between 12 and 45 years from both sexes, who were on oral antidiabetic treatment and insulin injection or combination therapy for a minimum of 6 months were included in the study (Figure 1). The patients were repeatedly visiting the Endocrine Clinic of the Persian Gulf Tropical Medicine Research Center, Bushehr, Iran, for routine medical examination at the time of the recruitment.

### 2.2. Inclusion Criteria

Patients with T1D, aged between 12 and 45 years and with good peripheral veins were included. Patients on insulin treatment were included as long as they were on stable doses and the dosage was not changed during the course of the study.

### 2.3. Exclusion Criteria

Subjects on any antioxidant therapy, such as vitamin supplements, having an allergy to grapes, green tea, and peanuts, and patients with T2D, severe heart disease, hepatic disease, renal dysfunction, and smoking habits were excluded.

### 2.4. Compliance

At the second and third visits, participants returned unused capsule bottles, and the study compliance was assessed by counting the remaining capsules from the bottle. Every participant was asked to complete a questionnaire during each visit to monitor the type of foods consumed during the study, particularly to confirm that they did not have food products that may have contained resveratrol. Moreover, at each visit, blood collection, weight measurement, and general medical examinations were done.

### 2.5. Treatment Regimen

All participants received 500 mg, twice daily (a total of 1 g/day), of resveratrol capsules (99% pure, Biotivia, Bioceuticals International, SRL, Verona, Italy) for a period of two months. All patients were allowed to continue their existing antidiabetic medications, including insulin injection, during the course of the study. Oral hypoglycemic agents and insulin were not modified during the course of the study. The participants who completed the study were on antidiabetic medications as follows (drug and number of patients on a particular drug): Insulin (13), Novarapid + Lantus + Insulin (5), Insulin + Metformin (1), Insulin + Glibenclamide (1), and Lotion Insulin (1). 

### 2.6. Physical Measurement

Height was measured with a stadiometer, and weight using a standard weighing balance. Outer garments and shoes were removed before measurements were done. Body mass index (BMI) was calculated as weight in kilograms divided by the square of height in meters using the Global Database on BMI (World Health Organization, 2006).

### 2.7. Biochemical Measurements

Fasting blood samples (12 hours) were taken from all participants at the baseline, after 30 days and 60 days of resveratrol treatment. All samples were promptly centrifuged, and the serum was separated and kept frozen at −80 °C until used. Analyses for biochemical parameters such as hemoglobin A1c (HbA1c), fasting blood sugar (FBS), liver enzymes, creatinine, albumin, and C-reactive protein (CRP) were carried out at the Persian Gulf Tropical Medicine Research Center on the day of blood collection by using a Selectra 2 autoanalyzer (Vital Scientific, Spankeren, The Netherlands). FBS levels were measured by using a commercial kit as per the manufacturer’s instructions (Pars Azmun Inc., Tehran, Iran). HbA1c levels were measured by using a commercial kit as per the manufacturer’s instructions (Nycocard HbA1c, Axis-SHIELD poc AS, *Axis*-*Shield* Group. P.O. Box 6863 Rodeløkka, N-0504, Oslo, *Norway*). Blood urea nitrogen, creatinine, albumin, and CRP levels were estimated by using a commercial kit as per the manufacturer’s instructions (Pars Azmun Inc., Tehran, Iran). Lipid peroxidation levels (Malondialdehyde—MDA) were measured by using a commercial kit as per the manufacturer’s instructions (Biomedica Medizinprodukte, GmbH and Co KG, Wien, Austria). Total antioxidant capacity (TAC) was measured by using a commercial kit as per the manufacturer’s instructions (Biovision Company, Milpitas, CA, USA). The levels of tumor necrosis factor-α (TNF-α) and interleukin-1β (IL-1β) were measured by using the commercial ELISA kits as per the manufacturer’s instructions (Karmania Pars Gene Company, Kerman, Iran). Serum insulin level was measured as per the manufacturer’s instructions (infinitumbiotech, 1935 Cordell Court El Cajon, CA, USA). In order to test liver function in the patients, activities of serum glutamic oxaloacetic transaminase (SGOT), serum glutamic pyruvic transaminase (SGPT), and alkaline phosphatase (ALP) were measured by using a commercial kit as per the manufacturer’s instructions (Pars Azmun Inc., Tehran, Iran).

### 2.8. Homeostasis Model of Assessment

Insulin resistance was assessed by calculating the homeostasis model of assessment index (HOMA-IR) using the following equation: Fasting insulin (μIU/mL) × fasting glucose (mg/dL)/405. The percentage of β-cell function from fasting serum glucose and insulin concentrations was assessed by calculating the homeostasis model of assessment index (HOMA-β) using the following equation: 360 × fasting insulin (μIU/mL)/fasting glucose (mg/dL) − 63 (4).

### 2.9. Statistical Analysis

The distribution of variables was determined using probability plots and the Shapiro–Wilk test. Data for HOMA-IR, HOMA-β, CRP, and insulin levels were log-transformed to obtain a Gaussian distribution. The anthropometric and biochemical parameters for patients with T1D that were assessed at baseline, 30th day, and 60th day after resveratrol supplementation (3-time points) were analyzed with repeated measures ANOVA. When sphericity assumptions were violated, degrees of freedom were adjusted using the Greenhouse Geisser correction. Pearson correlation analysis was used to study the relationships between HbA1c and the log-transformed HOMA-IR, HOMA-β, and insulin values. A post hoc test was done with Bonferroni correction. A *p* value of <0.05 was accepted as statistically significant. All statistical analyses were performed using PASW Statistics GradPack 18 (SPSS Inc., Chicago, IL, USA) and GraphPad Prism software, version 8.0.2 (GraphPad Software, Inc., San Diego, CA, USA).

## 3. Results

A total of 13 patients (8 males and 5 females) with T1D were evaluated at the endpoint in this trial. The mean age (mean ± SD) of the subjects was 23.61 ± 6.67 years. Apart from one patient who used insulin in combination with metformin, the entire study group received insulin at least two times per day throughout the study. No statistically significant changes were observed in the anthropometric measurements (body weight and BMI) of the patients from the baseline after resveratrol treatment (Table 1).

A repeated measure one way-ANOVA with Greenhouse–Geisser correction was done to detect differences from baseline to endpoint, and changes in the mean values of HbA1c between the three times (0 day, 30th day, and 60th day) of resveratrol supplementation were observed; (Table 1, 8.26 ± 0.97 vs. 8.02 ± 0.97 and 7.74 ± 1.02, F = 7.697, *p* = 0.009). There was a statistically significant decrease in levels of HbA1c at 60 days of resveratrol supplementation in comparison to the baseline levels (8.26 ± 0.97 vs. 7.74 ± 1.02, *p* = 0.033) before the commencement of treatment. However, the changes between the values of HbA1c at 30 days of resveratrol supplementation compared to baseline values were not significant (8.26 ± 0.97 vs. 8.02 ± 0.97, *p* = 0.080). There was also a significant decrease in the level of FBS after 60 days of resveratrol treatment in T1D patients in this study (253.69 ± 49.67 vs. 174.38 ± 45.19, *p* < 0.001). There were no significant differences in the levels of insulin, HOMA-IR, HOMA-β, and CRP between the three time points during the study (Table 1). The results of liver and kidney function tests (SGOT, SGPT, ALP, Albumin, BUN, and Cr) during the resveratrol supplementation period also did not demonstrate any significant changes (Table 1).

**Figure 2:**
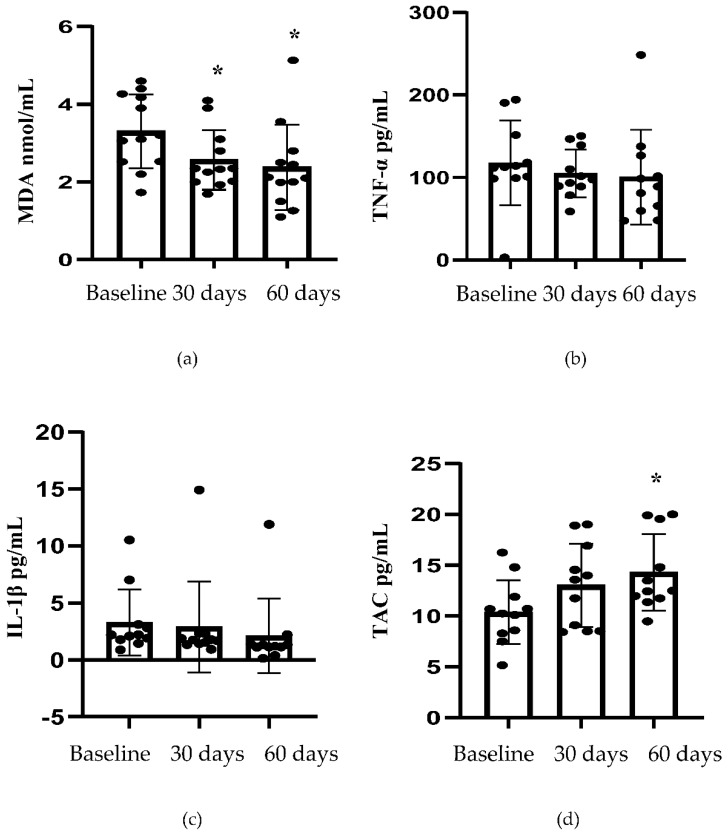
(**a**–**d**) show the levels of Malondialdehyde (MDA), tumor necrosis factor-α (TNF-α), interleukin-1β (IL-1β) and total antioxidant capacity (TCA) at different time points, in patients with T1D. Data are presented as means ± SD. Signs (*) on the top of each time point are indicators of significant *p* values (*p* < 0.05) of pairwise comparisons. The comparisons between the two time points and their significant values were assessed by using the post hoc Bonferroni test.

The differences in the levels of MDA and TAC in T1D patients were significant between the baseline and the endpoints (Figure 2, *p* < 0.002 and *p* < 0.001, respectively). There was no change in the serum levels of TNF-α and IL-1β between the three time points (Figure 2, *p* = 0.569 and 0.346, respectively). 

In bivariate correlation analysis, no significant correlations were found between the change in HbA1c values (end of the study period minus baseline) and the changes in HOMA-IR (r = 0.304, *p* = 0.313), HOMA-β (r = 0.075, *p* = 0.808), insulin (r = 0.180, *p* = 0.555), and FBS levels (r = −0.079, *p* = 0.797) during the study period. Moreover, significant correlations were found between the change in FBS levels (end of the study period minus baseline) and the changes in HOMA-β (r = −0.643, *p* = 0.018) during the study period. However, no significant correlation was observed between the change of FBS levels and the changes in insulin (r = −0.303, *p* = 0.315), and HOMA-IR (r = −0.351, *p* = 0.240).

## 4. Discussion

The primary objective of the current study was to undertake a preliminary investigation on the safety and efficacy of resveratrol in reducing FBS and HbA1c in T1D patients. In this exploratory clinical trial, we demonstrated for the first time that short-term treatment with resveratrol could decrease the levels of FBS and HbA1c in young adult T1D patients managed with insulin therapy. Furthermore, resveratrol reduced the level of the biomarkers for oxidant imbalance in T1D patients. The reduction in these parameters was achieved on top of the recommended insulin treatment without any adverse effects.

The current preclinical evidence suggests that resveratrol effectively reduces the levels of glucose and HAb1c by improving pancreatic β-cell function in different animal models of T1D [11,12,13,14,15,16,17]. The major factors contributing to T1D-related hyperglycemia are reduced or no insulin secretion, decreased glucose utilization by the peripheral tissue, and increased glucose production. Resveratrol has been shown to improve all of the factors mentioned above in the setting of T1D, as reported by previous preclinical studies [18,19,20,21,22]. In this current study, we also observed that resveratrol treatment significantly reduced FBS and HbA1c in T1D patients. This is consistent with our previous randomized clinical trial, which showed that treatment with resveratrol (500 g twice daily) for a duration of 45 days could significantly decrease the levels of FBS and HbA1c in T2D patients [23]. HbA1c is a highly sensitive and specific diagnostic biomarker used to detect prolonged hyperglycemia. It has also been reported to be significantly associated with an increase in diabetes complications. Presently, guideline-directed medical treatment of diabetes focuses on reducing the levels of HbA1c. Specifically, a reduction of 0.3% (3 mmol/mol) in the level of HbA1c has been recognized to be a clinically valuable target for achieving positive outcomes in terms of long-term diabetes-induced complications [24,25]. In this study, two-month resveratrol treatment resulted in a reduction of HbA1c by 5.7 mmol/mol from the baseline value. It should be noted that T1D patients in this study had high HbA1c values (8.26%) at baseline, which suggests that resveratrol treatment may be beneficial in difficult-to-manage T1D patients. Furthermore, a higher level of HbA1c is recognized as a significant risk factor for cardiovascular diseases and stroke in diabetics [26]. A HbA1c level of <7% (5.3 mmol/mol) may substantially reduce microvascular complications of diabetes patients. For example, large scale studies, namely, The Diabetes Control and Complications Trial (DCCT) and The United Kingdom Prospective Diabetes Study, established a linear correlation between HbA1c and microvascular abnormalities in diabetes patients [27]. Even though resveratrol treatment was not able to bring down HbA1c to <7% in T1D patients in this study, it is important to note that short-term resveratrol treatment resulted in a promising reduction of HbA1c (7.74%). In this study, HOMA-IR and β-cell function (HOMA-β) were not significantly reduced by resveratrol treatment in T1D diabetes patients unlike the significant decrease in both parameters by resveratrol in T2D patients reported in our previous study [23]. That being said, it should be noted that there was a trend towards a reduction in HOMA-IR and HOMA-β in the current study. 

Resveratrol-mediated decrease in HbA1c was also coupled with significant positive changes in the secondary outcomes of this trial. Inflammation and oxidative stress are key mechanisms underlying the genesis of T1D. Oxidative stress also plays a central role in the origin and advancement of diabetes-induced microvascular and macrovascular complications [28,29]. In this study, resveratrol treatment was associated with a decrease in the level of a marker for oxidative stress, MDA, in T1D patients. In addition, T1D patients showed an increase in the antioxidant defiance, as evidenced by an increase in the level of TAC. This finding suggests that resveratrol may offer additional protection and a better therapeutic profile as an adjuvant therapy. Our results are consistent with preclinical studies, which have reported that resveratrol reduced the level of oxidative stress markers and improved antioxidant defenses. A reduction in oxidative stress markers, such as superoxide anion, hydroxyl radical, hydrogen peroxide, MDA, 8-isoprostane, 8-hydroxydeoxyguanine, nitro-tyrosine, and reduced/oxidized glutathione [30,31,32,33,34], as well as an improvement in the activities of antioxidant enzymes, such as superoxide dismutase, catalase, glutathione peroxidase, and glutathione-S-transferase, has been observed in the setting of diabetes [33,35,36]. Resveratrol also attenuates autoimmune-mediated destruction of insulin-releasing pancreatic β-cells. This was demonstrated in nonobese diabetic mice, an animal model for T1D [37]. This animal model is characterized by diabetes that develops due to an autoimmune disease-related destruction of the functioning β-cells as the animals age [37]. In contrast to the significant reduction in oxidative stress observed in T1D patients with resveratrol treatment, there was no significant decrease/increase in the levels of proinflammatory markers, such as CRP, TNF-α, and IL-1β in this study. We also observed that there was no significant elevation of liver or kidney function biomarkers such as SGOT, SGPT, ALP, Albumin, BUN, and Cr in T1D patients. This suggested that resveratrol treatment was well tolerated in T1D patients. These results on the effects of resveratrol on liver or kidney function biomarkers were similar to those observed in our previous study in T2D patients [23].

It is also important to note that potential additional benefits may be achieved with resveratrol in T1D patients. Insulin resistance has also been identified as a complication in T1D patients. It is widely recognized that additional treatment may be needed in conjunction with insulin therapy to address the complication mentioned above [38]. As reported by the DCCT trial, the intensive insulin therapy resulted in a 33% increase in risk of being overweight with a mean weight gain of 4.6 kg over the treatment period. The term “double diabetes” has been coined to diagnose those individuals that are identified as T1D autoantibody positive and develop significant insulin resistance later in life [39]. To address this unique clinical difficulty, frontline T2D drug metformin has been explored as an addition to insulin treatment in such T1D patients [39]. Preclinical data showed that resveratrol was efficacious in preventing metabolic syndrome related to high-fat feeding, including insulin resistance, which lead to the pursuance of randomized clinical trials in T2D patients [40,41]. Evidently, resveratrol is known to recover dysregulated glucose homeostasis, while decreasing insulin resistance in T2D patients as reported by a myriad of clinical trials. These findings have been further verified by a few meta-analyses as well [23,42,43,44]. Specifically, a meta-analysis that reviewed 11 randomized controlled trials involving 388 subjects reported that resveratrol could reduce hyperglycemia and improve insulin sensitivity in diabetic patients, but could not affect levels of glucose in nondiabetic patients [43,45]. A second meta-analysis of clinical studies with resveratrol in T2D patients (nine randomized controlled trials with 283 subjects) reported that resveratrol could significantly reduce levels of FBS with concomitant beneficial effects on HOMA-IR scores and levels of insulin [42]. Very recently, a randomized clinical trial involving 71 overweight patients with T2D reported that eight-week resveratrol supplementation was able to significantly improve cardiac and metabolic parameters [46]. Furthermore, another recent randomized, double-blind, placebo-controlled trial with 56 patients having T2D and coronary artery disease reported that resveratrol reduced hyperglycemia and levels of MDA and improved the levels of HDL-cholesterol, the total-/HDL-cholesterol ratio, and TAC [47]. These promising clinical findings in patients with T2D may point towards a possible success of resveratrol in T1D patients as well.

There are some strengths and limitations associated with the current trial. Importantly, this is the first exploratory trial report of the short-term use of resveratrol for the treatment of T1D. The promising finding from an exploratory study may inform us about the possibility of designing rigorous long-term randomized trials in T1D patients. The important observations arising from our two clinical trials (involving T1D and T2D patients) reveal that resveratrol is not only able to complement the existing treatment, it may even offer additional protection over standard antidiabetic medications. A limitation of this study is that this was not a randomized, placebo-controlled, and double-blinded trial. However, this study provides preliminary safety and efficacy data on the potential of resveratrol in T1D. These results may form the basis of prospective randomized, placebo-controlled, double-blinded trials that can further ascertain the potential of resveratrol as an antidiabetic medication. It must be noted that the population of this study was Iranian, so the results of the current study may not be as reflective of the efficacy of resveratrol in patients of other ethnicities. The presence of potential confounding variables, such as varying ages of the patients, sex differences, and dissimilar antidiabetic treatments, may have also influenced the significance level of some of the parameters (which showed no significant difference). A comprehensive set of psychosocial parameters in relation to diabetes treatment is now being recognized as a set of highly imperative study endpoints for clinical trials as per the recommendations [48]. Future investigations on the efficacy of resveratrol in T1D patients may also explore such parameters. In this study, we did not follow up with patients to see if both of the reported parameters such as FBS and HbA1c, which showed significant reduction, were later reversed to the baseline levels with the discontinuation of resveratrol treatment. This is an aspect that can be explored in the future studies as well. Lastly, we did not undertake examination of in-depth mechanisms/pathways of action of resveratrol because this was an exploratory trial.

## 5. Conclusions

In conclusion, the addition of resveratrol to insulin therapy in T1D patients resulted in a significant and rapid reduction in the level of FBS with a concomitant reduction in HbA1c and oxidative stress. These observations warrant a detailed investigation as to the potential of resveratrol for the treatment of T1D in future prospective randomized, placebo-controlled, double-blinded trials.

## Figures and Tables

**Table 1:** Anthropometric and biochemical parameters in patients with type 1 diabetes (T1D) mellitus before and after resveratrol supplementation. Data is presented as means ± SD; BMI: Body mass index; FBS: Fasting blood sugar; HOMA-IR: Homeostasis model of assessment for insulin resistance; HOMA-β: Homeostasis model of assessment for β-cell function; HbA1c: Hemoglobin A1c; ALP: Alkaline phosphatase; SGOT: Serum glutamate oxaloacetate transaminase; SGPT: Serum glutamate pyruvate transaminase; BUN: Blood urea nitrogen. Cr: Creatinine.

	Baseline	30 Days	60 Days	F Value	*p* Value
Body weight (kg)	62.93 ± 12.45	63.57 ± 11.29	63.80 ± 11.29	1.80	0.201
BMI (kg/m^2^)	22.10 ± 3.35	22.38 ± 3.19	22.45 ± 3.16	2.46	0.135
FBS (mg/dL)	253.69 ± 49.67	199.92 ± 43.48	174.38 ± 45.19	18.27	<0.001
Insulin (µIU/mL)	14.72 ± 3.38	12.07 ± 2.14	14.96 ± 2.13	0.26	0.767
HbA1c	8.26 ± 0.97	8.02 ± 0.97	7.74 ± 1.02	7.69	0.009
HOMA-IR	13.20 ± 14.51	7.49 ± 6.84	5.54 ± 2.66	3.23	0.084
HOMA-β	71.43 ± 109.29	48.60 ± 53.55	54.44 ± 46.76	0.501	0.612
CRP (mg/dL)	5.22 ± 1.39	5.12 ± 1.35	4.66 ± 1.49	1.29	0.287
SGOT (IU/L)	19.38 ± 8.13	18.0 ± 7.83	18.07 ± 8.03	0.12	0.880
SGPT (IU/L)	17.69 ± 4.93	17.15 ± 10.93	15.61 ± 9.33	0.26	0.771
Albumin (gr/dL)	4.64 ± 0.37	4.63 ± 0.33	4.62 ± 0.34	0.02	0.980
ALP ((IU/L)	419.84 ± 390.54	392.769 ± 294.01	338.76 ± 293.31	1.50	0.243
BUN (mg/dL)	27.84 ± 7.40	25.30 ± 8.22	26.76 ± 6.69	0.69	0.509
Cr (mg/dL)	0.68 ± 0.33	0.59 ± 0.30	0.56 ± 0.34	2.39	0.120

## References

[1] Tuomilehto J. (2013). The emerging global epidemic of type 1 diabetes. Curr. Diabetes Rep..

[2] Maahs D.M., West N.A., Lawrence J.M., Mayer-Davis E.J. (2010). Epidemiology of type 1 diabetes. Endocrinol. Metab. Clin. N. Am..

[3] Dandona P., Mathieu C., Phillip M., Hansen L., Tschöpe D., Thorén F., Xu J., Langkilde A.M. (2018). Efficacy and Safety of Dapagliflozin in Patients With Inadequately Controlled Type 1 Diabetes: The DEPICT-1 52-Week Study. Diabetes Care.

[4] Miller K.M., Foster N.C., Beck R.W., Bergenstal R.M., DuBose S.N., DiMeglio L.A., Maahs D.M., Tamborlane W.V., Network T.D.E.C. (2015). Current state of type 1 diabetes treatment in the U.S.: Updated data from the T1D Exchange clinic registry. Diabetes Care.

[5] Seaquist E.R., Anderson J., Childs B., Cryer P., Dagogo-Jack S., Fish L., Heller S.R., Rodriguez H., Rosenzweig J., Vigersky R. (2013). Hypoglycemia and diabetes: A report of a workgroup of the American Diabetes Association and the Endocrine Society. Diabetes Care.

[6] Purnell J.Q., Zinman B., Brunzell J.D., Group D.E.R. (2013). The effect of excess weight gain with intensive diabetes mellitus treatment on cardiovascular disease risk factors and atherosclerosis in type 1 diabetes mellitus: Results from the Diabetes Control and Complications Trial/Epidemiology of Diabetes Interventions and Complications Study (DCCT/EDIC) study. Circulation.

[7] Conway B., Miller R.G., Costacou T., Fried L., Kelsey S., Evans R.W., Orchard T.J. (2010). Temporal patterns in overweight and obesity in Type 1 diabetes. Diabet. Med..

[8] Varanasi A., Bellini N., Rawal D., Vora M., Makdissi A., Dhindsa S., Chaudhuri A., Dandona P. (2011). Liraglutide as additional treatment for type 1 diabetes. Eur. J. Endocrinol..

[9] Van Gaal L.F., Wauters M.A., De Leeuw I.H. (1997). The beneficial effects of modest weight loss on cardiovascular risk factors. Int. J. Obes. Relat. Metab. Disord. J. Int. Assoc. Study Obes..

[10] Purnell J.Q., Hokanson J.E., Marcovina S.M., Steffes M.W., Cleary P.A., Brunzell J.D. (1998). Effect of excessive weight gain with intensive therapy of type 1 diabetes on lipid levels and blood pressure: Results from the DCCT. Diabetes Control and Complications Trial. JAMA.

[11] Oyenihi O.R., Oyenihi A.B., Adeyanju A.A., Oguntibeju O.O. (2016). Antidiabetic Effects of Resveratrol: The Way Forward in Its Clinical Utility. J. Diabetes Res..

[12] Fiori J.L., Shin Y.K., Kim W., Krzysik-Walker S.M., González-Mariscal I., Carlson O.D., Sanghvi M., Moaddel R., Farhang K., Gadkaree S.K. (2013). Resveratrol prevents β-cell dedifferentiation in nonhuman primates given a high-fat/high-sugar diet. Diabetes.

[13] Chen K.H., Cheng M.L., Jing Y.H., Chiu D.T., Shiao M.S., Chen J.K. (2011). Resveratrol ameliorates metabolic disorders and muscle wasting in streptozotocin-induced diabetic rats. Am. J. Physiol. Endocrinol. Metab..

[14] Gencoglu H., Tuzcu M., Hayirli A., Sahin K. (2015). Protective effects of resveratrol against streptozotocin-induced diabetes in rats by modulation of visfatin/sirtuin-1 pathway and glucose transporters. Int. J. Food Sci. Nutr..

[15] Simas J.N., Mendes T.B., Paccola C.C., Vendramini V., Miraglia S.M. (2017). Resveratrol attenuates reproductive alterations in type 1 diabetes-induced rats. Int. J. Exp. Pathol..

[16] Ku C.R., Lee H.J., Kim S.K., Lee E.Y., Lee M.K., Lee E.J. (2012). Resveratrol prevents streptozotocin-induced diabetes by inhibiting the apoptosis of pancreatic β-cell and the cleavage of poly (ADP-ribose) polymerase. Endocr. J..

[17] Palsamy P., Subramanian S. (2008). Resveratrol, a natural phytoalexin, normalizes hyperglycemia in streptozotocin-nicotinamide induced experimental diabetic rats. Biomed. Pharmacother..

[18] Li Y., Huang J., Yan Y., Liang J., Liang Q., Lu Y., Zhao L., Li H. (2018). Preventative effects of resveratrol and estradiol on streptozotocin-induced diabetes in ovariectomized mice and the related mechanisms. PLoS ONE.

[19] Chen T.S., Kuo C.H., Day C.H., Pan L.F., Chen R.J., Chen B.C., Padma V.V., Lin Y.M., Huang C.Y. (2019). Resveratrol increases stem cell function in the treatment of damaged pancreas. J. Cell. Physiol..

[20] Arrick D.M., Sun H., Patel K.P., Mayhan W.G. (2011). Chronic resveratrol treatment restores vascular responsiveness of cerebral arterioles in type 1 diabetic rats. Am. J. Physiol. Heart Circ. Physiol..

[21] Szkudelska K., Szkudelski T. (2010). Resveratrol, obesity and diabetes. Eur. J. Pharmacol..

[22] Su H.C., Hung L.M., Chen J.K. (2006). Resveratrol, a red wine antioxidant, possesses an insulin-like effect in streptozotocin-induced diabetic rats. Am. J. Physiol. Endocrinol. Metab..

[23] Movahed A., Nabipour I., Lieben Louis X., Thandapilly S.J., Yu L., Kalantarhormozi M., Rekabpour S.J., Netticadan T. (2013). Antihyperglycemic effects of short term resveratrol supplementation in type 2 diabetic patients. Evid. Based Complement. Altern. Med..

[24] Lind M., Polonsky W., Hirsch I.B., Heise T., Bolinder J., Dahlqvist S., Schwarz E., Ólafsdóttir A.F., Frid A., Wedel H. (2017). Continuous Glucose Monitoring vs Conventional Therapy for Glycemic Control in Adults With Type 1 Diabetes Treated With Multiple Daily Insulin Injections: The GOLD Randomized Clinical TrialContinuous Glucose Monitoring for Glycemic Control in Type 1 DiabetesContinuous Glucose Monitoring for Glycemic Control in Type 1 Diabetes. JAMA.

[25] Lind M., Oden A., Fahlen M., Eliasson B. (2010). The shape of the metabolic memory of HbA1c: Re-analysing the DCCT with respect to time-dependent effects. Diabetologia.

[26] Sherwani S.I., Khan H.A., Ekhzaimy A., Masood A., Sakharkar M.K. (2016). Significance of HbA1c Test in Diagnosis and Prognosis of Diabetic Patients. Biomark. Insights.

[27] Miller K.M., Beck R.W., Bergenstal R.M., Goland R.S., Haller M.J., McGill J.B., Rodriguez H., Simmons J.H., Hirsch I.B., Network T.D.E.C. (2013). Evidence of a strong association between frequency of self-monitoring of blood glucose and hemoglobin A1c levels in T1D exchange clinic registry participants. Diabetes Care.

[28] Domínguez C., Ruiz E., Gussinye M., Carrascosa A. (1998). Oxidative Stress at Onset and in Early Stages of Type 1 Diabetes in Children and Adolescents. Diabetes Care.

[29] Ceriello A. (2003). New Insights on Oxidative Stress and Diabetic Complications May Lead to a “Causal” Antioxidant Therapy. Diabetes Care.

[30] Palsamy P., Subramanian S. (2011). Resveratrol protects diabetic kidney by attenuating hyperglycemia-mediated oxidative stress and renal inflammatory cytokines via Nrf2-Keap1 signaling. Biochim. Biophys. Acta.

[31] Mohammadshahi M., Haidari F., Soufi F.G. (2014). Chronic resveratrol administration improves diabetic cardiomyopathy in part by reducing oxidative stress. Cardiol. J..

[32] Hamadi N., Mansour A., Hassan M.H., Khalifi-Touhami F., Badary O. (2012). Ameliorative effects of resveratrol on liver injury in streptozotocin-induced diabetic rats. J. Biochem. Mol. Toxicol..

[33] Schmatz R., Perreira L.B., Stefanello N., Mazzanti C., Spanevello R., Gutierres J., Bagatini M., Martins C.C., Abdalla F.H., Daci da Silva Serres J. (2012). Effects of resveratrol on biomarkers of oxidative stress and on the activity of delta aminolevulinic acid dehydratase in liver and kidney of streptozotocin-induced diabetic rats. Biochimie.

[34] Sedlak L., Wojnar W., Zych M., Wygledowska-Promienska D., Mrukwa-Kominek E., Kaczmarczyk-Sedlak I. (2018). Effect of Resveratrol, a Dietary-Derived Polyphenol, on the Oxidative Stress and Polyol Pathway in the Lens of Rats with Streptozotocin-Induced Diabetes. Nutrients.

[35] Kitada M., Kume S., Imaizumi N., Koya D. (2011). Resveratrol improves oxidative stress and protects against diabetic nephropathy through normalization of Mn-SOD dysfunction in AMPK/SIRT1-independent pathway. Diabetes.

[36] Soufi F.G., Mohammad-nejad D., Ahmadieh H. (2012). Resveratrol improves diabetic retinopathy possibly through oxidative stress–nuclear factor κB–apoptosis pathway. Pharmacol. Rep..

[37] Szkudelski T., Szkudelska K. (2015). Resveratrol and diabetes: From animal to human studies. Biochim. Biophys. Acta.

[38] Priya G., Kalra S. (2018). A Review of Insulin Resistance in Type 1 Diabetes: Is There a Place for Adjunctive Metformin?. Diabetes Ther..

[39] Cleland S.J., Fisher B.M., Colhoun H.M., Sattar N., Petrie J.R. (2013). Insulin resistance in type 1 diabetes: What is ‘double diabetes’ and what are the risks?. Diabetologia.

[40] Zhao H., Song A., Zhang Y., Shu L., Song G., Ma H. (2019). Effect of Resveratrol on Blood Lipid Levels in Patients with Type 2 Diabetes: A Systematic Review and Meta-Analysis. Obesity.

[41] Timmers S., Hesselink M.K., Schrauwen P. (2013). Therapeutic potential of resveratrol in obesity and type 2 diabetes: New avenues for health benefits?. Ann. N. Y. Acad. Sci..

[42] Zhu X., Wu C., Qiu S., Yuan X., Li L. (2017). Effects of resveratrol on glucose control and insulin sensitivity in subjects with type 2 diabetes: Systematic review and meta-analysis. Nutr. Metab. (Lond.).

[43] Liu K., Zhou R., Wang B., Mi M.T. (2014). Effect of resveratrol on glucose control and insulin sensitivity: A meta-analysis of 11 randomized controlled trials. Am. J. Clin. Nutr..

[44] Bhatt J.K., Thomas S., Nanjan M.J. (2012). Resveratrol supplementation improves glycemic control in type 2 diabetes mellitus. Nutr. Res..

[45] Hausenblas H.A., Schoulda J.A., Smoliga J.M. (2015). Resveratrol treatment as an adjunct to pharmacological management in type 2 diabetes mellitus--systematic review and meta-analysis. Mol. Nutr. Food Res..

[46] Abdollahi S., Salehi-Abargouei A., Toupchian O., Sheikhha M.H., Fallahzadeh H., Rahmanian M., Tabatabaie M., Mozaffari-Khosravi H. (2019). The Effect of Resveratrol Supplementation on Cardio-Metabolic Risk Factors in Patients with Type 2 Diabetes: A Randomized, Double-Blind Controlled Trial. Phytother. Res. PTR.

[47] Hoseini A., Namazi G., Farrokhian A., Reiner Z., Aghadavod E., Bahmani F., Asemi Z. (2019). The effects of resveratrol on metabolic status in patients with type 2 diabetes mellitus and coronary heart disease. Food Funct..

[48] Beck R.W., Lawrence J.M., Laffel L., Wysocki T., Xing D., Huang E.S., Ives B., Kollman C., Lee J., Ruedy K.J. (2010). Quality-of-life measures in children and adults with type 1 diabetes: Juvenile Diabetes Research Foundation Continuous Glucose Monitoring randomized trial. Diabetes Care.

